# Pharmaceutical Trace Analysis

**DOI:** 10.6028/jres.093.031

**Published:** 1988-06-01

**Authors:** D. Scott Aldrich, Steven J. Borchert, Amy Abe, James E. Freeman

**Affiliations:** The Upjohn Company, Kalamazoo, MI 49001

Trace analysis can have various meanings, depending on the context of the problem at hand. This paper focuses on one aspect of trace analysis relevant to quality control in the pharmaceutical industry. Trace levels of contaminants in pharmaceutical materials and products can present significant analytical challenges in many ways. For example, organic impurities in drugs and drug products are typically controlled to levels from 0.01–1.0%. Detecting and quantitating species at these levels represents a complex problem in mixture analysis. The development of such methods frequently requires the analysis of overlapped chromatographic peaks and chemically labile species. For compounds of acute toxicological concern, part-per-billion (ppb) levels must sometimes be monitored. Apart from toxicology issues, some chemical contaminants present at a few ppm can produce undersirable color or odor in a product.

Although the above examples represent everyday problems in pharmaceutical trace analysis, the focus of this paper is in another area: the detection, identification, and control of trace levels of insoluble material in drug solutions. Insoluble materials can occur as individual particles visible to the eye, visually perceptible haze, or subvisible particles. Some medical evidence of the consequences of particulate matter entering the bloodstream does exist [1–3]. Furthermore, various compendial regulations limit the numbers and sizes of particles allowed in parenteral drug products [4–6]. For example, no particles visible to the eye may be present. Recent trends within the pharmaceutical regulatory community indicate increasingly restrictive requirements pertaining to particulate matter.

The particle size range of interest for pharmaceuticals is 5–100 μm. The lower limit is determined by the size of capillary blood vessels, and the upper limit is the threshold of visible detection. That this type of contamination is a trace analytical problem can be appreciated from the data in table 1. The concentrations of particles fall in the ppm range, and typically as little as a few nanograms of material must be detected and analyzed.

Because particulate matter must be controlled on a routine basis, the best approach to dealing with such problems is in the formulation and manufacturing design stages. We have followed this strategy: (1) detection and problem definition, (2) isolation and sample preparation, (3) identification, and (4) elimination of the mechanism of particle formation [7]. The following discussion includes brief descriptions of each of these points and examples of actual problems we have encountered.

The characteristics of human and instrumental particle-detection technology have been thoroughly described in the literature ([7], and references therein). Two alternatives exist for detecting “visible particles,” i.e., particles > 100 μm. Human visual inspection, while sensitive and selective as to its ability to classify particle types, suffers from inspector fatigue and poor reproducibility. This process has been shown to be probabilistic, not deterministic: it cannot detect visible particles with 100% certainty. Machine visual inspection is more reproducible and possesses a lower particle-size detection limit. Subvisible particles must not only be detected, but also sized and counted. A number of instrumental approaches to this problem have been developed, but the method based on detecting and sizing particles by their ability to block a light beam has been almost universally adopted by the pharmaceutical industry.

Proper definition of the problem as, for instance, individual large particles, haze, etc. is important for the next steps, which involve isolation of the particles and preparing them for analysis. Commonly, only a small number of product containers with only a few particles are available for analysis. The most common isolation techniques are filtration and single-particle isolation by micropipette. The surface on which particles are isolated can determine what subsequent analytical techniques can be applied. We have found the use of gold-coated membrane filters to be particularly useful when surface-sensitive spectroscopic techniques are to be used.

Microscopy is invariably the first technique applied to particle analysis: microscopy is sensitive, with a detection limit of one picogram, and it is ideally suited to assessing the heterogeneity of the isolated particles. Light microscopy techniques can determine refractive index, crystal form, solubility, thermal properties, and some chemical properties. Elemental analysis (scanning electron microscopy/x-ray fluorescence, ESCA), molecular spectroscopy (infrared, Raman, mass spectrometry), and other techniques (light scattering, x-ray diffraction, chromatography) must frequently be used to completely identify particulate matter. Importantly, SEM/XRF, Raman, and infrared spectroscopy can each be used to analyze nondestructively single particles.

Identification includes not only chemical identity, but also the source of the particles. Particles can arise from the manufacturing environment, packaging materials, or the drug solution itself. The examples described below illustrate two such sources. Once the mechanism of particle formation is understood, steps to eliminate it from the product design can be taken.

In the first example, large visible particles were detected in a diluent solution used for reconstituting lyophilized drug powders. The solution contained water, methyl and propyl paraben, and sodium pantothenate. Microscopy revealed a heterogeneous matrix, one component of which was identified by infrared spectroscopy as silicone oil. SEM/XRF analysis revealed the presence of Cu and S; the remainder of the particles was organic in nature. Using x-ray fluorescence and atomic absorption spectroscopy, Cu was quantitated in solution at about 1 ppm and traced to the lot of sodium pantothenate used for these preparations. Product manufactured from other lots of sodium pantothenate containing lower levels of Cu did not produce a significant amount of precipitate.

Copper alone could not account for the formation of the precipitate, and the presence of silicone oil implicated the lubricated rubber stoppers. A series of experiments demonstrated that both the presence of Cu and contact with the rubber stoppers used in the product were necessary to form particles. Subsequent mass spectral analysis of the particles confirmed the presence of a common vulcanizing agent, 2-mercaptobenzothiazole (MBT). MBT is known to be extracted from rubber by contact with aqueous solutions, and its reaction with Cu yields a highly insoluble complex [8–10]:

$$\text{Cu}+2\text{MBT}=\text{Cu}{\left(\text{MBT}\right)}_{2},K_{\text{sp}}=10^{-21}.$$

Experiments confirming the Cu concentration dependence of particle formation, as well as identification of the individual components, were important to discovering this mechanism. An obvious solution to this problem is the use of stoppers which do not contain MBT.

In the second example, particles were observed as a fine “smoke” in a new product under development. Interestingly, the smoky character of the particles prevented their detection by machine inspection; only careful human inspection revealed their presence. Microscopically, these particles were amorphous flakes 10–30 μm in size. SEM/XRF analysis did not detect elements of atomic number >9. ESCA detected significant amounts of only C and O, and high-resolution measurements suggested the presence of an ester functional group. Infrared analysis detected carbonyl and C–O–C stretching frequencies characteristic of aromatic esters, and Raman spectroscopy confirmed the presence of aromatic functionality with a band at 1000 cm^−1^. Mass spectrometry revealed several series of repeating mass fragments and, hence, the polymeric nature of the particles. A detailed analysis of the infrared and mass spectra (figs. 1 and 2), followed by analysis of specially synthesized authentic material, confirmed the identity of the particles as poly(diethyleneglycol)isophthalate. This material was subsequently traced to filtration equipment used during synthesis of the drug.

These examples illustrate the wide range of techniques which can be used to identify particulate matter. Detection and resolution of such problems contributes significantly to the design and manufacture of high-quality pharmaceutical products.

## Figures and Tables

**Figure 1:**
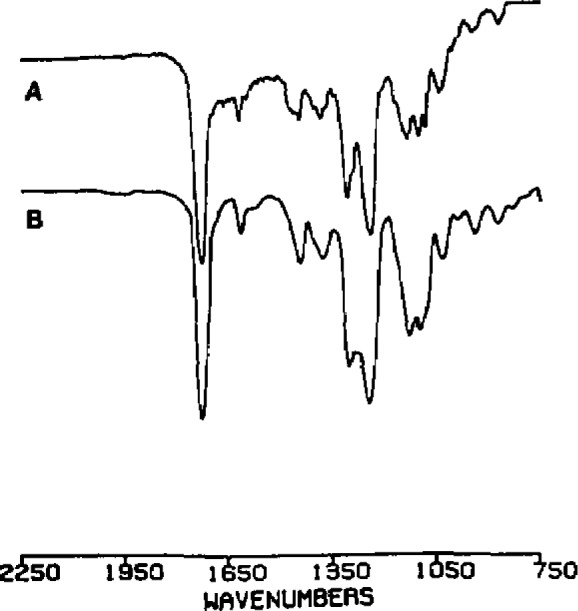
Infrared spectra of (A) particles and (B) synthetic poly(diethyleneglycol)isophthalate.

**Figure 2:**
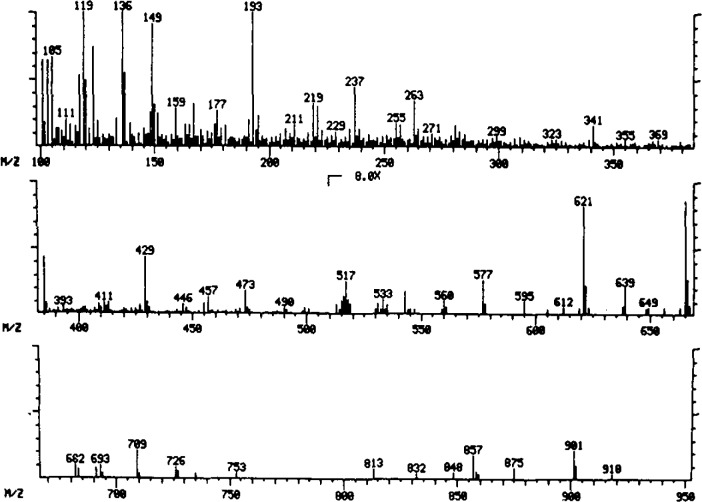
Desorption chemical ionization mass spectrum of particles.

**Table 1:** Mass and concentration of 1 g/cm^3^ spherical particles at various regulatory limits

Limit	Particle mass per container (μm)	Particle concentration (ppb)
One 100-μm particle per 10-mL container	0.52	52.
1000 2-μm particles per mL	0.0042	4.2
100 5-μm particles per mL	0.0065	6.5
50 10-μm particles per mL	0.026	26.
5 25-μm particles per mL	0.041	41.
